# Editorial to the IJMS Special Issue on “ncRNAS in Therapeutics”

**DOI:** 10.3390/ijms24076448

**Published:** 2023-03-29

**Authors:** Miguel Hueso, Estanis Navarro

**Affiliations:** 1 Department of Nephrology, Hospital Universitari de Bellvitge, 08907 L’Hospitalet de Llobregat, Spain; 2 Nephrology and Renal Transplantation Group, Infectious Disease and Transplantation Program, Institut d’Investigació Biomèdica de Bellvitge-IDIBELL, 08907 L’Hospitalet de Llobregat, Spain; 3 Independent Researcher; Esplugues de Llobregat, 08950 Barcelona, Spain; estanis.navarro@gmail.com

For many years, the RNA world of eukaryotic cells remained stable and predictable, organized by a few families of functionally different molecules. Thus, ribosomal RNAs structured the translational machinery; hnRNA/mRNAs transmitted the genetic information from nucleus to the cytoplasm; and a plethora of small RNAs (tRNAs, snRNAs, snoRNAs) played additional structural or regulatory roles in translation, splicing, etc. In this context, the DNA/RNA sequencing revolution at the turn of the XXIth century revealed a surprising fact, namely that the RNA world (also known as the RNAome) was far richer and more complex than anticipated. In fact, in 2007 the ENCODE pilot project demonstrated that more than 90% of the human genome was pervasively transcribed [1], producing a stunning number of non-coding RNAs (ncRNAs), either short or long, that could be differentiated by their size, subcellular localization, and function. As a consequence, the number of RNA families has grown to include new elements such as miRNAs, eRNAs, circRNAs, AS-RNAs, sponge-RNAs, etc., many of them associated with human diseases [2]. The functional richness of these ncRNAs offers the possibility of using them in disease therapies by overexpressing or downregulating ncRNAs whose expression is altered in human diseases.

This Special Issue, entitled “ncRNAS in Therapeutics”, focuses on this important topic of research, providing examples of this use of ncRNAs, as well as on the vehicles used for specific RNA delivery to cells and tissues. In this context, De Benedittis and cols. [3] performed a pilot study on the value of six microRNAs (miRNAs) as biomarkers of drug resistance in temporal lobe epilepsy, demonstrating that three of them (miR-142, miR-146a, miR-223) were significantly upregulated in drug-resistant patients and could be used as diagnostic circulating molecules of resistance to drug treatment. This is particularly interesting because a large number of patients with TLE are refractory to drug treatment with antiseizure medications (ASMs). As such, so there is a clear need for the early detection of responding patients. The authors concluded that expression analysis of circulating miR-142 and miR-223 could be useful to distinguish drug-sensitive vs. drug-resistant TLE patients. Following this line of inquiry in the miRNA field, Di Fiore and cols. [4] reviewed current data on the utility of miRNA determination for the diagnosis and treatment of rare gynaecological tumours associated with poor prognosis. These authors reported on miRNAs whose expression is altered in uterine tumours (sarcomas and carcinosarcomas), vulvar carcinomas, malignant melanomas of the female genital tract, gestational trophoblastic disease (GTD), and ovarian cancers (epithelial and nonepithelial). Furthermore, they deepened the investigation into their putative targets to give a mechanistic view of the oncogenic processes.

On the lncRNA side, Kuwara and cols. [5] discussed on ncRNAs (lncRNAs and miRNAs) with a role in the processes of myocardial infarction and cardiac remodelling after myocardial infarction, and also considered the preclinical therapies targeting these ncRNAs and their potential toxic effects. On the other hand, Aurilia and cols. [6] reviewed the lncRNAs involved as positive or negative regulators in the processes of osteoblastogenesis, osteoclastogenesis and bone tumourigenesis as well as the molecular mechanisms in which they are involved. Their aim was to detect new biomarkers for the early diagnosis of bone-related disorders and for the development of new therapeutic strategies.

This Special Issue also includes two technical notes. Lee and cols. [7] reviewed the preparation of circRNAs, with the aim of circumventing the stability problems usually associated with RNAs and, consequently, increasing their translation, a critical point for their use as therapeutic agents or in vaccine preparation. The authors gave a complete view of the use of chemical-, enzyme- or ribozyme-based methods for circularizing RNAs, and highlighted current limitations that must be overcome in order to popularize the use of this interesting tool. On the other hand, Hueso and cols. [8] discuss the problems hindering the use of ncRNAs in the clinical setting, and more specifically those associated with the tissue-specific delivery of the RNA molecules, ways to increase their stability, and the “on-target” and “off-target” side effects. Essentially, they aimed to advance (i) the development of improved algorithms for a more efficient hybridization to targets, (ii) the development of new chemistries for stabilizing RNAs, and (iii) the development of more efficient vehicles for specific targeting, centring on the use of nanoparticles as delivery vehicles. Lastly, the authors also discussed the problems facing the clinical use of nucleic acids for ncRNA therapy. This special issue gives a glimpse of the complexity of the RNAome and its possible uses for the treatment of human diseases. It is sure that the years to come will see an spectacular growth of new approaches and technological developments to facilitate the use of RNAs as therapeutic tools against human diseases. 
